# Supplementary figures and images for: One-pot Golden Gate Assembly of an avian infectious bronchitis virus reverse genetics system
Source: PLoS One. 2024 Jul 25;19(7):e0307655. doi: 10.1371/journal.pone.0307655 (PMC11271894; doi:10.1371/journal.pone.0307655)

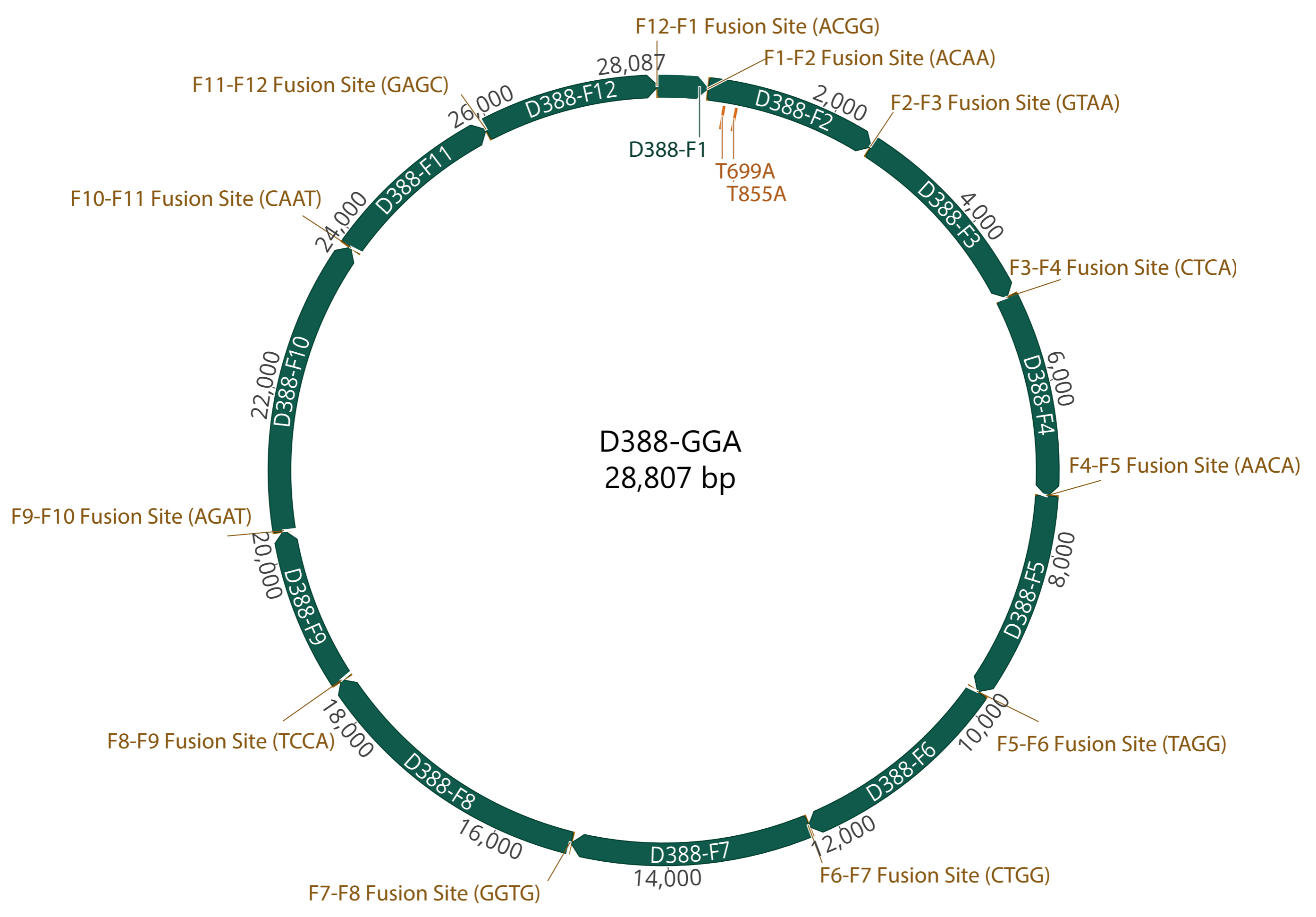

Supplement: S1 Fig — A Genbank file of the full genome with the annotations is also provided as part of the Supporting Information. (PDF) [file pone.0307655.s001.pdf]
