# Supplementary material for: One-pot Golden Gate Assembly of an avian infectious bronchitis virus reverse genetics system
Source: PLoS One. 2024 Jul 25;19(7):e0307655. doi: 10.1371/journal.pone.0307655 (PMC11271894; doi:10.1371/journal.pone.0307655)
Supplement: S2 Fig — Data-optimized Assembly Design tools (ligasefidelity.neb.com) were used to check the fidelity of the assembly. The predicted ligation fidelity for the overhangs used in all IBV Golden Gate Assemblies (ACGG, ACAA, GTAA, CTCA, AACA, TAGG, CTGG, GGTG, TCCA, AGAT, CAAT, GAGC) is 97%. The reaction conditions used for this prediction were “BsaI-HFv2 37–16 cycling”. (PDF) [file pone.0307655.s002.pdf]

Estimated ligation fidelity: 97%

Using the given set of overhangs, Golden Gate Assembly is predicted to yield 97% of correctly-ligated products.

Ligation frequency matrix

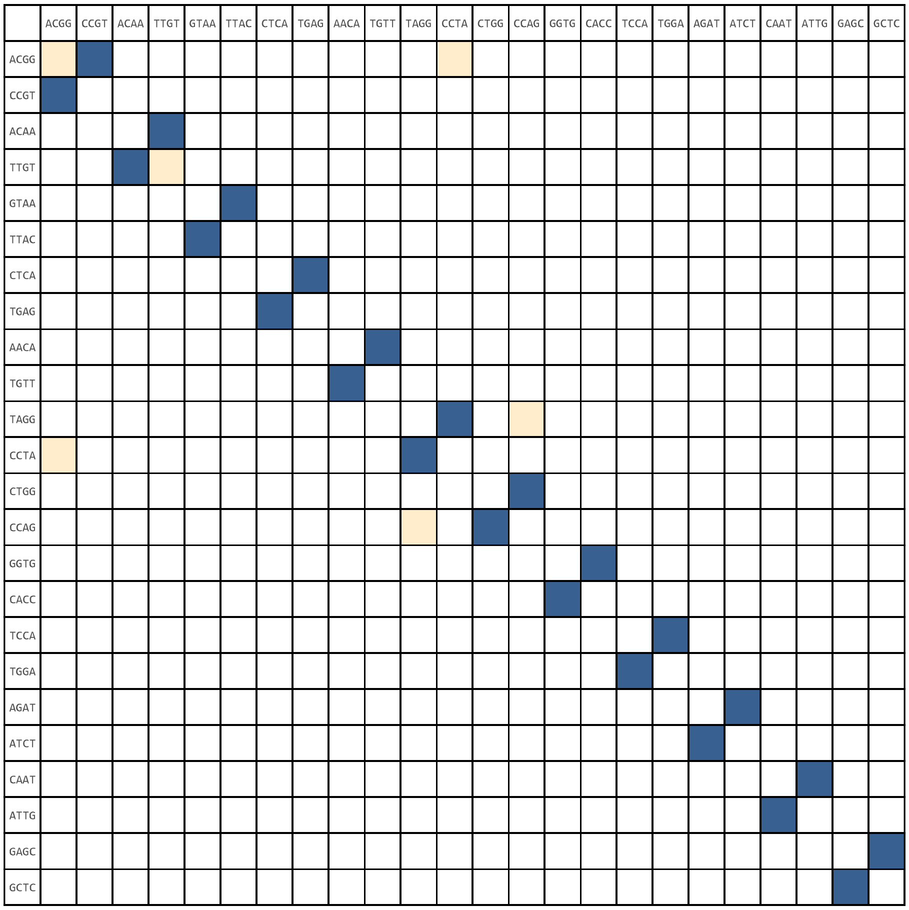

Legend

- good Watson-Crick pair
- poor Watson-Crick pair
- high-count mismatch
- modest mismatch
- trace mismatch
