# Supplementary material for: One-pot Golden Gate Assembly of an avian infectious bronchitis virus reverse genetics system
Source: PLoS One. 2024 Jul 25;19(7):e0307655. doi: 10.1371/journal.pone.0307655 (PMC11271894; doi:10.1371/journal.pone.0307655)
Supplement: S3 Fig — Assembly success was optionally assessed prior to rescue via visualization on TapeStation, a gel electrophoresis system that can visualize fragments up to ~40kb. The TapeStation molecular weight ladder is in lane 1. An example of pre- and post-assembly samples is shown in lanes 2 and 3, respectively. The assembly mastermix (lane 2) shows a range of sizes that reflects the 12 input vector parts. The shortest fragment (D388-F1) is 2451 bp and all other fragments range from 3741 bp to 5338 bp. Higher molecular weight species in lane 2 represent non-supercoiled populations of the input parts. Following the assembly reaction, the assembled D388-GGA cDNA (28 kb) (lane 3) runs between the two highest standards (15000 bp and 48500 bp). Under the cycling conditions used, the majority of the DNA is seen in this peak, with a second peak at 1.9 kb that is the empty vector backbone cut away from the fragments during assembly. (DOCX) [file pone.0307655.s003.docx]

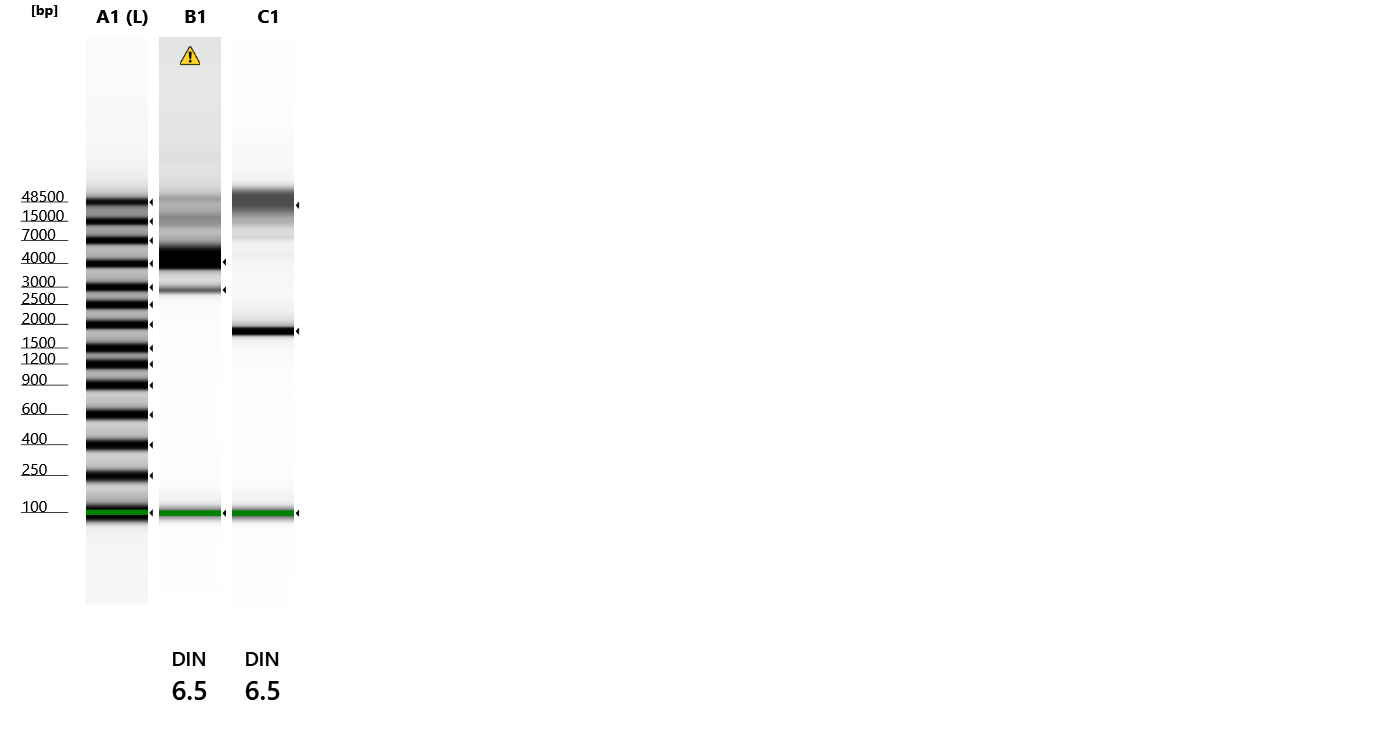
**Figure S3. Tapestation gel to evaluate D388-GGA assembly reaction.**

3

2

1

bp

Empty Donor Vectors

D388-GGA

Input Vectors
