# Supplementary material for: One-pot Golden Gate Assembly of an avian infectious bronchitis virus reverse genetics system
Source: PLoS One. 2024 Jul 25;19(7):e0307655. doi: 10.1371/journal.pone.0307655 (PMC11271894; doi:10.1371/journal.pone.0307655)
Supplement: S1 Raw images — (PDF) [file pone.0307655.s007.pdf]

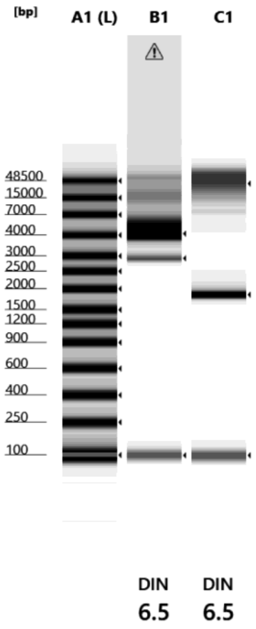

Original TapeStation Data for Fig S3

Lane A1 Ladder
Lane B1 IBV D388-GGA Master Mix
Lane C1 IBV D388-GGA Assembled

Default image (Contrast 100%), Image is Scaled to Sample

Sample Info

Well	DIN	Conc. [ng/µl]	Sample Description	Alert	Observations
A1	-	57.6	Ladder		Ladder
B1	6.5	124	IBV D388-GGA Master Mix	⚠	Sample concentration outside recommended range
C1	6.5	52.1	IBV D388-GGA Assembled		
