# Supplementary material for: One-pot Golden Gate Assembly of an avian infectious bronchitis virus reverse genetics system
Source: PLoS One. 2024 Jul 25;19(7):e0307655. doi: 10.1371/journal.pone.0307655 (PMC11271894; doi:10.1371/journal.pone.0307655)
Supplement: Attachment — Submitted filename: Response to Reviewers.docx [file pone.0307655.s009.docx]

5. Review Comments to the Author

Please use the space provided to explain your answers to the questions above. You may also include additional comments for the author, including concerns about dual publication, research ethics, or publication ethics. (Please upload your review as an attachment if it exceeds 20,000 characters)

Reviewer #1: This research did not yield noteworthy findings regarding the current investigation into IBV. Instead, it presents a novel methodology better suited for journals emphasizing new protocols or methods. Notably, it highlights previously introduced point mutations using a distinct approach.

Response to reviewer: We respectfully disagree with the reviewer’s comment that our research has not yielded noteworthy findings. Our manuscript details the establishment of a one-pot reverse genetics system that permits the easy manipulation of the IBV genome on a greatly accelerated timeline compared to traditional methods. This presents a valuable tool for the continued study of this economically important pathogen. We further note that the PLOS ONE submission guidelines state the following: “PLOS ONE considers Research Article submissions which report new methods, software, databases and tools as the primary focus of the article. These should also adhere to the utility, availability and validation criteria in the guidelines for specific study types.” Upon reviewing the specific guidelines for a methods study in PLoS One, we strongly believe that our manuscript supports the utility, availability, and validation criteria as outlined.

Introduction:
• The claim that "Coronaviruses are a family of viruses that infect a wide variety of species and that share a large (~30kb) positive sense RNA genome structure" is untrue. Coronaviruses mostly affect birds and mammals, not a large range of species. Furthermore, the size of the genome differs between coronaviruses, ranging from roughly 26 to 32 kilobases (kb), rather than a constant 30 kb.

Response to reviewer: The text has been changed to reflect the reviewers comment that coronaviruses infect birds and mammals. The “~” symbol has been changed to the word “approximately.”

• The introduction explains that "Avian infectious bronchitis virus (IBV), a gammacoronavirus and the first coronavirus identified, is the etiological agent of Infectious Bronchitis (IB)." Even though IBV is a gammacoronavirus, it wasn't the first one to be discovered. The mouse hepatitis virus was the first coronavirus to be identified in 1949. The human coronavirus OC43 was discovered in the 1960s.

Response to reviewer: As indicated in the text, Reference 1 describes the discovery of the Beaudette strain of infectious bronchitis virus in 1937, which predates the discovery of mouse hepatitis virus in 1949. The infectious disease, Infectious Bronchitis (IB), was first described in 1931 (Schalk et al) with the cultivation of the associated infectious agent, IBV described in 1937. This is widely accepted in the field and additionally referenced in Nature Reviews Microbiology (V’kovski et al 2021) in which a timeline of notable events in the field is presented – both this reference and the reference describing IB in 1931 have been added to the statement.

• There should be more specificity added to the phrase, "Many IBV strains cause classical respiratory disease characterised by snicking, tracheal rales, watery eyes, nasal discharge, lethargy, reduced weight gain, and drops in egg production and quality."

Response to reviewer: We are unsure of what additional specificity the reviewer would like to see included beyond the list of symptoms but would be happy to add additional references if any can be suggested.

• Regarding reverse genetics, the introduction mentions, "Classic reverse genetics systems for coronaviruses were first developed in the early 2000s based on RNA recombination in vivo, in vitro ligation using pre-existing or uniquely engineered restriction sites, or assembly and propagation of a cDNA copy of the coronavirus genome in a Bacterial Artificial Chromosome (BAC) or Vaccinia Virus Vector." While some reverse genetics systems were indeed established in the early 2000s, the field of reverse genetics for coronaviruses dates back further, with significant advancements made in the 1990s.

Response to reviewer: We apologize to the reviewer that our statement was incomplete and have added more detail. The first reverse genetics system that allowed the modification of a full-length cDNA copy of a coronavirus genome was published in 2000 by Almazan *et al*, and was based on transmissible gastroenteritis (TGEV). We have added this reference to the manuscript and clarified the text to read “Classic reverse genetics systems enabling the modification of a full-length cDNA copy of the coronavirus genome were first developed in the early 2000’s...” Prior to that the reviewer is correct in that advancements were made using RNA recombination; however, this method did not permit recovery of infectious clones from full length cDNA nor did it allow for the easy manipulation of Nsps, which account for two thirds of the genome. We have added the following to further acknowledge the advancements made in the 1990s - “The first method of modifying the coronavirus genome was described in the 1990s and was based on RNA recombination. It was originally based on Murine Hepatitis Virus (MHV) using temperature sensitive lesions within the N gene. While this was a significant advancement, the technology was limited in application and did not easily allow for modification of all areas of the genome; it was therefore was not widely adopted for all coronaviruses”. We have also added an appropriate reference to this section.

• The introduction talks about how a reverse genetics system for the betacoronavirus SARS-CoV-2 was developed using the Golden Gate Assembly (GGA). Nevertheless, the connection between this and the gammacoronavirus IBV and its reverse genetics system is not made clear.

Response to reviewer: We have updated this sentence:

“ Golden Gate Assembly (GGA) is widely used in synthetic biology to create systems for studying gene networks and metabolic pathways, and was recently applied to develop a new reverse genetics system for SARS-CoV-2^37^.”

to the following:

“ Golden Gate Assembly (GGA) is widely used in synthetic biology to create systems for studying gene networks and metabolic pathways, and was recently applied to develop a new reverse genetics system for another coronavirus, the betacoronavirus SARS-CoV-2^37^.”

Methods:
• The exact concentrations of reagents used in various steps, such as RNA extraction, Golden Gate Assembly reactions, and cell culture inoculation, are not provided.

Response to reviewer: We have added additional detail to the following sections of the Methods:

Determination of the genomic 5′ end sequence– We have added the quantity of virus and the RNA extraction kit used. The line now reads:

“RNA was extracted from 170 µl of the stock of D388-WT using a RNeasy mini kit (Qiagen) following the manufacturers method for RNA cleanup”

Analysis of IBV-derived RNA in harvested tissues- We have added the quantity of RNA added to the RT-qPCR reaction. The section now reads:

“The Luna Universal Probe One-Step RT-qPCR kit (NEB) following the manufacturer’s protocol was used for the quantification of IBV derived RNA using IBV specific primers (forward 5′-GCTTTTGAGCCTAGCGTT-3′ and reverse 5′-GCCATGTTGTCACTGTCTATTG-3′) that target the 5′ UTR alongside the probe 5-6-carboxyfluorescein (FAM)-CACCACCAGAACCTGTCACCTC-6-carboxytetramethylrhodamine (TAMRA)-3′. Each reaction contained 500 ng of RNA.”

We disagree with the reviewer regarding the detail for the Golden Gate assembly. The procedure is explained in full detail in the section “Assembly of IBV D388 cDNA” (see below):

“For all assemblies, each part was quantitated in triplicate using a Qubit assay (Thermo Fisher) and an equimolar fragment master mix was prepared to ensure a mole-balanced mixture of fragments. GGA reactions (20 µL final volume) were carried out with 3 nM of each DNA fragment from the master mix and 2 µL of the NEBridge Golden Gate Assembly Kit (BsaI-HFv2) (NEB E1601S) in 1X T4 DNA Ligase Reaction Buffer. Reactions were cycled between 37°C and 16°C for 5 minutes each for 90 cycles, followed by a final 60°C heat soak for 5 min, before being stored at -20°C until virus recovery experiments. 90 cycles were used to maximize yield of full length assembly product.”

Similarly, we disagree about the methods for cell culture inoculation, which we presume means the quantity of virus used to infect cells or ex vivo TOCs. These methods are detailed in the following sections. For the response to reviewers comments we have underlined and put in bold the virus inoculation details.

**Assessment of ciliary activity in *ex vivo* tracheal organ cultures (TOCs).**

TOCs were seeded one per tube and only those with 100% ciliary activity were used for the experiment. In replicates of at least ten, TOCs were inoculated with 1 ml TOC infection media (0.5 x EMEM, 75 mM a-methyl-D-glycoside, 40 mM HEPES, 0.1% sodium bicarbonate, 10 U/mL penicillin, 10 mg/mL streptomycin) containing **10^3^ CD_50_ of either D388 (WT), D388-GGA, D388-14mt-GGA, D388-10.14mt-GGA, D388-Beau(S)-GGA** or 1 ml media only for mock infection. TOCs were incubated at either 37 or 41°C. Using a light microscope, the ciliary activity of each TOC was assessed at 24 h intervals and graded as follows: 0, 25, 50, 75 or 100 % activity as based on previously published protocols^48,82^.

**Assessment of replication *in ovo***

In replicates of three or five, 10-11 day-old SPF embryonated hens’ eggs were inoculated with **10^3^ CD_50_** IBV/rIBV and incubated at 37°C for 24 h. Eggs were chilled for at least 4 h at 4°C after which the allantoic fluid was harvested. The quantity of infectious progeny present in the allantoic fluid was determined via titration in *ex vivo* TOCs.

**Analysis of IBV Infection in CK and Vero Cells by Confocal Microscopy.**

CK and Vero cells were seeded onto glass coverslips in 24 well tissue culture plates at 0.8 x 10^6^ and 0.4 x 10^5^ cells/ml, respectively, 3 days prior to infection. On the day of infection, cells were washed once in Phosphate-buffered saline a (PBSa) and inoculated with D388-WT, D388-GGA, D388-Beau(S)-GGA or Beau-R (a molecular clone of Beau-CK)^36^ at an **MOI of > 1** in a volume of 200 µl.

• The sequencing procedure is not explained clearly enough. It does not specify which software tools or techniques were utilised for the assembly process, even though it claims using a "in-house pipeline" to put sequencing reads together.

Response to reviewer: The protocol used was identical to that already published in the indicated reference (Freimanis and Oade, 2020). We have however edited the section to include additional details of the pipeline. The section now reads:

RNA was prepared from 2ml of allantoic fluid that compromised the stock virus of D388-WT and the recombinant IBV (rIBV) D388-GGA following a previous published protocol^46^. As previously published, library preparation for all samples was performed in triplicate with 100 ng total RNA for each sample, using a NEBNext directional Ultra II RNA-Seq kit (NEB, Ipswich, MA). Library QC was performed using the Bioanalyzer 2100 DNA 1000 kit and Qubit BR kit, prior to pooling. All libraries were sequenced on an Illumina MiSeq, using a MiSeq reagent v3 600 cycle cartridge. Analysis was performed using an in-house pipeline. Briefly, Trim Galore (http://www.bioinformatics.babraham.ac.uk/projects/trim_galore/) and fastqc (https://www.bioinformatics.babraham.ac.uk/projects/fastqc/) were used to perform initial read QC and quality filtering with minimum quality scores of 30 and minimum read length of 100 nucleotides. Host subtraction was performed by mapping all reads to the chicken genome (*Gallus gallus*) (GCA_000002315.5) using BWA-MEM^77^. All remaining reads were then subjected to a de novo assembly using SPAdes^78^. The resulting contigs were scaffolded using reference genome MN548289^79^ for D388-WT and the D388-WT sequence for D388-GGA. Reads were aligned back onto the *de novo* reference sequences and the resulting alignment was used to generate a consensus level reference sequence using BWA-MEM^77^. All nucleotide positions referenced in this manuscript relate to the deposited D388-WT sequence, GenBank accession number OR813926, unless otherwise stated.

• The name used to refer to IBV is inconsistent throughout the section; for example, it is called both "Infectious Bronchitis Virus" and "Infectious Bronchitis virus." It would be easier to read and understand if the terminology was standard.

Response to reviewer: We fixed inconsistent capitalization throughout the text.

Results:
• While the study describes the design of the reverse genetics system in detail, it lacks thorough validation of the design process. More information on the rationale behind specific design choices and experimental verification of the designed sequences would enhance the credibility of the approach.

Response to reviewer: The design approach used here reflects our broader design approach, described in even more detail in the provided references (Potapov *ACS Syn Bio* 2018, Pryor *PLOS ONE* 2020, Pryor *ACS Synthetic Biology* 2022, Sikkema *Current Protocols in Molecular Biology* 2023). We have described the design for this specific assembly target in detail in the Results subsection “Design and Assembly of a GGA-based reverse genetics system for avian IBV.” For example, to explain the choice to divide the genome into 12 fragments, the following is already stated in the manuscript: “The sequence was rationally divided into 12 fragments (Fig. 1, Supplementary Fig. 1, Supplementary Table 2) generally restricting the fusion sites to the regions between ORFs, with an additional fusion site placed at each end of the genome as the circularization fusion site. This rational breakpoint strategy ensured that sequences of interest were grouped together or isolated into a single fragment to allow for easy manipulation and interrogation of individual genes and proteins. As an example of the strategy considerations, the S gene encoding the spike protein, which is essential to study for antiviral and vaccine development, is contained in its entirety on fragment D388-F10 (Fig. 1).” The rest of this section describes the rationale behind other design choices. If the reviewer has a specific question about aspect(s) of the design rationale we would be open to addressing it.

With regard to the reviewer’s comment about experimental verification of the design sequences, we have made note in the methods that all assembly parts were ordered as sequence-verified plasmids from Genscript.

We additionally note that the rescued virus is characterized by sequencing and the only mutations present are the silent mutations we incorporated to remove native Type IIS sites (“Next Generation Sequencing confirmed that the sequence of the D388-GGA IBV genome matched the target design with no mutations beyond the two intentionally introduced silent mutations.”) As detailed in the manuscript the replication of the recovered viruses are characterized *ex vivo* and *in ovo*, and where appropriate *in vivo*.

• The introduction of silent mutations for marker purposes is a common approach; however, the study does not provide experimental evidence to confirm that these mutations do not affect viral replication or phenotype. Including functional assays to demonstrate the lack of impact of these mutations on viral fitness would strengthen the validity of the approach.

Response to reviewers: The incorporation of marker mutations is vital when designing reverse genetics system to ensure that the molecular clone and wild type virus can be differentiated. The wild type (WT) virus and the molecular clone generated in this study, D388-GGA replicate comparably both *in ovo* and *in vivo* as described in Figure 2 and Figure 3. This would therefore suggest that the marker mutations in Nsp 2 have not affected viral replication, particularly in the natural host. We also additionally note in the results section that the marker mutations were present in viral RNA extracted from D388-GGA infected birds therefore suggesting that the mutations are stably maintained and therefore not negatively impacting viral replication. The line in the results section reads: “Of note, the presence of the marker silent mutations in nsp2 that differentiate D388-WT from D388-GGA were confirmed in IBV RNA isolated from tracheal tissue, ensuring that observed results were as a result of infection by the assembly-derived IBV.” We do note in the manuscript that there is a difference in phenotype in primary chicken kidney cells which through the generation of another rIBV is attributed to the Spike gene and not to the marker mutations in Nsp 2 (Figure 5). The exact residues involved regarding *in vitro* tropism are out of the scope of this manuscript. We have added the following to the discussion:

“In further support, D388-GGA exhibited comparable replication to the wild type virus both *in ovo*, specifically at 24 hpi, and *in vivo* (Fig. 2 and Fig. 3) suggesting that the marker mutations had not negatively impacted viral replication in the natural host. Sequencing of RNA recovered from *in vivo* infected tissues demonstrated the marker mutations had been retained, further suggesting that the marker mutations had not negatively impacted viral replication *in vivo*.”

• While the study characterizes the replication kinetics of the recombinant viruses in vitro and in vivo, it lacks detailed analysis of key viral properties such as antigenicity, virulence, and host immune response. Additional experiments, such as serological assays or animal challenge studies, would provide a more comprehensive understanding of the behaviour of the recombinant viruses.

Response to reviewer: The purpose of our study was to establish a reverse genetics system that allowed rapid and easy modification to the IBV genome. The rIBVs generated in the study were made as a proof of principle on the ease of use of the system, and what modifications could be made, including whole gene replacements and point mutations. The further examination of the phenotype of the generated rIBVs, including vaccine-challenge studies, is out of scope of this particular manuscript but is being followed up as part of other projects. In terms of serological assays for the *in vivo* study presented in this manuscript, the birds were culled seven days post infection and at this time point (and the earlier time points of four- and six-days post infection) the antibody response is not yet developed. With regards to the broad term of host immune responses, the purpose of the *in vivo* study was to establish whether the molecular clone of the wild type virus could result in similar clinical disease and viral replication, which it does. The evaluation of unspecified host immune responses is out of scope of this manuscript.

• The study could benefit from including more controls, such as viruses with unrelated mutations or wild-type viruses, to better interpret the observed phenotypic differences between the recombinant and wild-type viruses.

This study is a methodology-focused paper and the first in ongoing work utilizing this reverse genetics system. As the reviewer notes, we wanted to provide sufficient detail of the design principles and method, as well as experimental validation of the resulting rIBVs. As stated above, the rIBVs generated in the study were made as a proof of principle on the ease of use of the system, and what modifications could be made, including whole gene replacements and point mutations. Ongoing follow up studies will address the connection between specific virus genotypes and resulting phenotypes in more detail. Finally, we note that the appropriate control was always included for each experiment, whether it was wild type IBV or a mock.

Reviewer #2: Katharina Bilott et al. present a novel one-pot reverse genetics system leveraging Golden Gate Assembly for the construction of the avian infectious bronchitis virus (IBV), a significant advancement in vaccine development. This methodology streamlines the generation of point mutants and gene replacements, offering a more efficient alternative to traditional methods. The IBV genome was segmented into 12 fragments, synthesized and amplified within E. coli plasmids, followed by in vitro assembly, showcasing the potential for creating point mutants and gene replacements. The manuscript is commendably structured, introducing an innovative and effective approach for IBV study and manipulation.

Response to reviewer: We thank the reviewer for their comments and are delighted that the reviewer recognizes that the manuscript is well structured and details an innovative and effective approach for the continued study of IBV.

Specific points,
1. A comparison highlighting the advantages and disadvantages of this IBV construction method relative to other reverse genetics approaches would enrich the discussion. Such analysis could provide a clearer understanding of the method's uniqueness and practicality.

Response to the reviewer: We thank the reviewer for this suggestion, and we have added a section (starting with “While prior vaccinia virus-based reverse genetics systems….) to the discussion detailing factors that need to be considered for coronavirus reverse genetics and how the system described here is advantageous. We hope that this has enriched the discussion.

2. The efficiency of recovering recombinant IBV remains unclear. Additional details on the recovery rates and comparative efficiency with traditional methods could underscore the method's effectiveness.

Response to reviewer: The recovery of rIBV is dependent on several factors, of which one is likely the efficiency of the assembly reaction. However, one of the main problems in IBV research is the inability for most IBV strains to infect and replicate in continuous cell lines; therefore, the recovery of rIBV is carried out in primary chicken kidney (CK) cells as they are supportive of IBV replication. As our well-established rescue protocol is carried out in primary cells, a significant factor that affects recovery of rIBV is the preparation and isolation of these cells and both their infectability and transmissibility. The rescue protocol requires three reagents to enter a single cell, the rFPV-T7, the assembly reaction, and the N plasmid, and it also requires the cell to be permissive for IBV replication. The primary CK cells are a mixed population and not every cell in the tissue culture dish is going to be permissive. Failure to successfully recover a rIBV is therefore not necessarily indicative of failure of assembly. We therefore feel that is not appropriate to draw conclusions on the recovery rate of rIBV; the primary focus of the study is to describe a new method of genome assembly using the Golden Gate technology. We have however added details to the Results (subsection “Recovery of recombinant IBV D388-GGA from transfection of assembled cDNA”) detailing that the recovery of the full-length clone (D388-GGA) was completed in triplicate and all three replicates resulted in successful recovery, thereby demonstrating that the assembly method was compatible with the existing viral recovery system:

“All three replicates were positive for IBV derived RNA with one taken forward for the generation of stock virus, demonstrating that the assembly method was compatible with the existing well-established method for rIBV recovery.”

3. On line 210, it is mentioned that "Next Generation Sequencing confirmed the sequence of the D388-GGA IBV genome". It would be beneficial to present these results, detailing the accuracy and fidelity of the assembled genome.

Response to reviewer: With regards to the accuracy and fidelity of the assembled genome, our NGS sequencing was completed on the recovered recombinant IBV and not the assembled genome. As stated in the manuscript the consensus sequence matched the target design with no mutations identified, however we have clarified that the NGS sequencing was completed on stock virus. Accuracy and fidelity of the assembled genome matched the designed sequence. We have clarified that the two intentionally introduced silent mutations are present. We have added information regarding number of reads and the coverage depth of NGS sequencing. The results text now reads:

“Next Generation Sequencing of the stock virus confirmed that the sequence of the D388-GGA IBV genome matched the target design; with no mutations were identified. The presence of the two intentionally introduced silent mutations to differentiate D388-GGA from D388 wild type was also confirmed. Next generation sequencing generated 1099234 reads which covered 100% of the genome with an average coverage depth of 9487.86.”

4. In line 544, the manuscript describes direct cell transfection with assembly reaction products without prior amplification or purification. Clarification is needed on how completeness and accuracy of the products are ensured without verification, potentially through product analysis or validation steps.

Response to reviewer: Typical of all cloning reactions, sequence verification of the assemblies is completed on the passaged products, not on the pre-transformed samples. Direct sequencing of the raw assembly reactions is complicated by the high molar concentration of empty vector backbone from the assembly parts that results post-assembly. We have updated Figure S3, which depicts a Tape Station gel containing samples before and after assembly, to demonstrate the accumulation of higher molecular weight IBV cDNA upon completion of the assembly reaction. Additionally, sequence verification was completed on rescued virus, as noted in the text. Of all virus populations picked and sequenced, all isolates were 100% sequence correct. We do also note that if the resulting virus did have any mutations that we would not necessarily be able to attribute them to the assembly reaction, as they could represent the natural accumulation of mutations during viral replication.

As described in answers to Reviewer 1, the sequenced virus isolated from the chickens retains the marker mutations, and the lack of reversion indicates that the mutations are not a fitness burden. This has also been added to the text.

5. The manuscript would benefit from an assessment of the stability and concentration of D388. Exploring the challenges in assembling multiple fragments, especially identifying the most problematic aspects, would provide valuable insights into the method's robustness and limitations.

The challenges of assembling multiple fragments have been explored in previous papers, including several from the Lohman lab. We note that the cited Current Protocols paper (Sikkema 2023) provides a comprehensive review of complex assembly, including methods, challenges, and troubleshooting.

Reviewer #3: The study employed Golden Gate Assembly design and synthetic biology techniques to construct an efficient reverse genetics system for Infectious Bronchitis Virus (IBV). Demonstrating the system's application in generating point mutants and gene replacements, the team validated its feasibility and flexibility. These findings suggest that the reverse genetics system based on Golden Gate Assembly is a reliable and efficient tool for researching the biological characteristics of IBV and for vaccine development.
The results were well organized and presented, which is suitable for publication in this journal. However, some issues should be revised before publication.

Response to reviewer: We thank the reviewer for their positive comments and we hope we have added sufficient information to address their comments.

Major:
1.The authors claimed no replication in the CK cells were observed by detecting the dsRNA with antibody, I wonder if this result has been confirmed by other available methods, eg: using specific antibodies against IBV N protein (which I think is more sensitive than detecting the dsRNA) or qPCR assay to detect sgmRNA at different time points.

Response to reviewer: We assessed replication of the D388-GGA clone in CK cells by several methods which included serially passaging the virus, plaque assays and RT-PCR. We were unable to passage the virus and CK cells infected with D388-GGA were negative for IBV derived RNA by RT-PCR, demonstrating that the virus could not establish an infection. Our attempts to titrate the virus via plaque assay also yielded no plaque further indicating that it could not replicate in CK cells. We have added the following to the Results subsection “Characterization of D388-GGA”:

“This was further supported through additional assays including RT-PCR analysis of D388-GGA infected CK cells in which no IBV derived RNA could be detected, plaque assays in which no viral plaques could be observed, and via serial passaging in which no virus could be detected.”

2.The replication characteristics of the D388-WT and D388-GGA were compared in 10-day old embryo chicken eggs at only one time point-24 hpi, which can not fully demonstrate the replication characteristics, more time points should be added in this assay to draw an in ovo growth kinetics.

Response to reviewers: In our experience, beyond 24 h the D388 strain of IBV causes embryos to die. We do not feel additional time points beyond this time point offer sufficient information to outweigh the ethical considerations. We have clarified the text to state that replication *in ovo* resulted in equivalent titers at 24 hpi. We have removed any text that states that replication is comparable *in ovo*.

This includes the following lines in the introduction and discussion, respectively:

“The resulting recombinant IBV (rIBV), denoted D388-GGA, was characterized and compared to D388-WT. Replication was comparable *in vivo* and comparable titers were achieved *in ovo* at 24 hours post infection (HPI).”

“The replication of both mutation-containing rIBVs *in ovo* resulted in titers comparable to D388-GGA at 24 hpi, with all rIBVs exhibiting titers of over 10^6^ CD50/ml (Fig. 4A and 4C).”

Minor:
1.L239: The specific infection dose of IBV in CK cells should be added as in 2A and 2B.

The cells were infected with MOI of > 1. The dose of IBV has been added to the figure legend.

2.L241: The presentation of the phrase ‘mouse double stranded RNA (dsRNA) and rabbit anti-tubulin’ is incomplete, the canonical phrase should be ‘mouse double-stranded RNA (dsRNA) monoclonal antibody’.

The text has been changed to reflect the correct phrase.

3.L496: Is nsp2 a separate ORF?

The original sentence

Silent mutations were required to remove two native BsaI sites in the nsp2 ORF and were selected using a codon table for *Gallus gallus* (T735A and T891A) (Supplementary Table 1).

has been changed as follows:

Silent mutations were required to remove two native BsaI sites in nsp2 (part of ORF 1ab) and were selected using a codon table for *Gallus gallus* (T735A and T891A) (Supplementary Table 1).

4.L579: The unit was missing behind 103.

Response to reviewer: We apologize that this was missing. We have added the unit.

5.The page number were missing in more than one references.

Some references do not have page numbers as they are online-only publications, some of which do not provide page numbers. Any articles where page numbers could be added have been fixed.
